# Phenotypic shifts induced by environmental pre-stressors modify antibiotic resistance in *Staphylococcus aureus*

**DOI:** 10.3389/fmicb.2023.1304509

**Published:** 2023-12-04

**Authors:** Gui Nam Wee, Eun Sun Lyou, Susmita Das Nishu, Tae Kwon Lee

**Affiliations:** Department of Environmental and Energy Engineering, Yonsei University, Wonju, Republic of Korea

**Keywords:** environmental stressors, phenotypic shift, biomolecular markers, Raman spectroscopy, antibiotic resistance

## Abstract

**Introduction:**

Escalating prevalence of antibiotic resistance in *Staphylococcus aureus* has necessitated urgent exploration into the fundamental mechanisms underlying antibiotic resistance emergence, particularly in relation to its interaction with environmental stressors. This study aimed to investigate the effects of environmental stressors prior to antibiotic exposure on the antibiotic resistance of *S. aureus*.

**Methods:**

We used Raman spectroscopy and flow cytometry to measure prior stress-induced phenotypic alterations of *S. aureus*, and identified the association between phenotypic shifts and the antibiotic resistance.

**Results:**

The results revealed a multifaceted relationship between stressors and the development of antibiotic resistance. The stressors effectuate distinct phenotypic diversifications and subsequently amplify these phenotypic alterations following antibiotic treatments, contingent upon the specific mode of action; these phenotypic shifts in turn promote the development of antibiotic resistance in *S. aureus*. This study’s findings demonstrated that the presence of pre-stress conditions triggered an augmentation of resistance to vancomycin (VAN), while concurrently attenuating resistance to norfloxacin. Marked shifts in Raman peaks associated with lipids and nucleic acids demonstrated correlations with elevated survival rates following VAN treatment.

**Conclusion:**

Consequently, these observations indicate that pre-stress conditions “prime” bacterial cells for differential responses to antibiotics and bear significant implications for formulating clinical therapeutic strategies.

## 1 Introduction

*Staphylococcus aureus* is an opportunistic pathogen that serves as the etiological agent of a diverse array of infections within its host organisms. The adaptability and resilience of *S. aureus* are predominantly predicted by its capacity for antibiotic resistance, a trait conjectured to stem from its exceptional ability to adjust promptly to alterations in environmental conditions (Machado et al., 2021; Matuszewska et al., 2022). The escalating prevalence of antibiotic resistance poses an increasing concern by rendering therapeutic strategies ineffective, thereby imposing considerable burdens and financial implications on individuals and healthcare systems (Poudel et al., 2023). Upon colonizing a host, *S. aureus* is invariably subjected to a range of environmental fluctuations, which frequently induce alterations in the phenotypic traits associated with its defense mechanisms (Poudel et al., 2023). This phenomenon posits that *S. aureus*, when confronted with antibiotic treatment, may not be in defenseless condition but could potentially be pre-armed with antibiotic resistance-inducing mechanisms that are already activated and poised for action (Huemer et al., 2021). Consequently, the antibiotic susceptibility of *S. aureus* may exhibit unpredictable variations, as it targets phenotypes modified by prior environmental conditions. Current *in-vitro* antibiotic susceptibility tests, which rely on axenic cultures grown in rich media, lack ecological validity. Therefore, investigating the influence of environmental stress conditions on the phenotype of *S. aureus* and its antibiotic susceptibility is extremely important to comprehensively elucidate the knowledge gap with urgent clinical implications.

Microorganisms undergo various phenotypic alterations in response to environmental stimuli and stressors, which encompass modifications in cell size, cellular complexity, efflux pumps, and membrane structure, and consequently induce the development of antibiotic resistance (Sánchez-Romero and Casadesús, 2014; Frenoy and Bonhoeffer, 2018). Starvation instigates the preservation of the transmembrane proton motive force, thereby amplifying tolerance to β-lactam antibiotics in both gram-negative and gram-positive bacteria (Wang et al., 2021). Microbial interactions possess the capacity to elicit specific phenotypic alterations in lipid profiles, such as d-alanylation of teichoic acids and lysylation of phosphatidylglycerol, as a means to repulse cationic antimicrobial peptides (Otto, 2009). Furthermore, fluctuations in the NaCl concentration have been observed to accelerate biofilm formation in *S. aureus*, which further contributes to an augmentation of antibiotic resistance (Alreshidi et al., 2020). Access to detailed phenotypic information prior to antibiotic treatment can substantially improve the predictive accuracy of antibiotic efficacy. Consequently, this presents a pressing need for research focused on linking phenotypic variability to antibiotic resistance.

The acquisition of sufficiently high-resolution phenotypic data for the elucidation of subtle modifications warrants the employment of laser-based single-cell phenotyping technologies. Raman spectroscopy and flow cytometry (FCM) are advanced phenotyping techniques used to investigate phenotypic variations in single cells. FCM is used to analyze cell size, complexity, and nucleic acid content at the single-cell level (Barr et al., 2021). These simple phenotyping resources provide intuitive and robust outcomes that are sensitive to environmental fluctuations, and demonstrate sensitivity to a variety of environmental stressor, such as antibiotics (O’Brien-Simpson et al., 2016; Andrade et al., 2020). Biomolecular phenotyping through Raman spectroscopy has emerged as a valuable approach for obtaining elaborate phenotypic profiles (Hong et al., 2021b). Raman spectra illustrate that biomolecular data, encompassing lipids, nucleic acids and proteins, provide extremely sensitive phenotypic data that can be correlated with gene expression in response to environmental stimuli. The aforementioned techniques have demonstrated considerable potency in antibiotic research, revealing that phenotypic changes attributable to environmental stressors can markedly affect antibiotic susceptibility. Therefore, the adoption of these sophisticated methodologies is indispensable for a thorough analysis of the topic at hand.

The primary objective of this research was to rigorously investigate the effects of environmental stressors prior to antibiotic exposure on the antibiotic resistance of *S. aureus*. Additionally, this study endeavored to identify the phenotypic variations associated with the escalation in antibiotic resistance. This can be accomplished by employing a combination of high-resolution phenotyping techniques, including FCM and Raman spectroscopy, to generate exhaustive phenotypic information. Bacterial cells were treated with physical, chemical, and biological stressors to induce phenotypic alterations in *S. aureus* prior to antibiotic treatment. After exposing the cells to stress conditions, they were examined for antibiotic susceptibility and phenotypic variations were traced for associations with antibiotic resistance. Therefore, this study aimed at bridging the extant knowledge gap pertaining to phenotypic shifts induced by environmental stressors and antibiotic susceptibility in anticipation of significantly advancing the scientific basis underlying microbial adaptive evolution and offering tangible benefits in clinical settings where predicting antibiotic susceptibility is critical for effective treatment strategies.

## 2 Materials and methods

### 2.1 Cultivation with stressors

The strains of *Staphylococcus aureus* NCTC 8325–4, *Staphylococcus epidermidis* American Type Culture Collection (ATCC) 12228, *Acinetobacter johnsonii* ATCC 17909, and *Micrococcus luteus* ATCC 4698 were procured from Chung-Ang University (Anseong, Korea) and the Korean Collection for Type Cultures (Jeongeup, Korea). The aforementioned strains, which were designated standard skin microbiome references by the ATCC, were chosen to examine microbial interactions with *S. aureus* owing to their cohabitation as skin microbiota. *S. aureus* and *S. epidermidis* were cultured in tryptic soy broth (TSB; BD, Franklin Lakes, NJ, United States) and incubated at 37°C and 120 rpm for 24 h. *A. johnsonii* and *M. luteus* were grown in TSB at 30°C and 120 rpm for 24 h. Following cultivation, the cultures were centrifuged at 15,928 × g for 3 min at 4°C, washed twice with TSB, and utilized for subsequent experiments.

To assess the impact of stress on the resistance of *S. aureus* to subsequent antibiotic treatments, this study examined four distinct stressors that *S. aureus* may encounter in the skin environment: microbial competition, starvation, salinity, and low pH. To acquire intact *S. aureus* cells subjected to microbial competition, a membrane-based co-culturing system was used that facilitated the physical segregation of the co-cultivated cells. Bacteria were cultured in 24-mm Transwell plates equipped with a 0.4-μm polyester membrane (Corning Inc., Corning, NY, United States) at 30°C and gently agitated at 25 rpm for 12 h to aid the diffusion of metabolites between the different compartments. *S. aureus* cell suspensions (3.0 mL) were loaded onto the plates and 3.0 mL each of *S. epidermidis* (+SE), *A. johnsonii* (+AJ), and *M. luteus* (+ML) were aliquoted into the inserts. The initial cell densities were calibrated to be 10^6^ cells/mL for each strain. To subject the bacteria to the other three stressors, starvation, salinity, and low pH, *S. aureus* were cultured in the Transwell plates in TSB lacking 0.05% glucose-containing dextrose (−Glu), in TSB at pH 4 (+HCl), and in TSB supplemented with 5% NaCl (+NaCl), respectively, and gently agitated at 25 rpm at 37°C for 12 h. Controls were incubated under identical conditions without any treatment. The co-cultures or cultures exposed to stressors were prepared in triplicates and randomized over plates to account for plate effects.

### 2.2 Antibiotic susceptibility change test

The susceptibility of *S. aureus* NCTC 8325–4 to three different antibiotics possessing distinct modes of action (Table 1) was evaluated at their minimum inhibitory concentrations. The antibiotic stock solutions were prepared by dissolving vancomycin (VAN) in distilled water, chloramphenicol (CHL) in ethanol, and norfloxacin (NOR) in glacial acetic acid. Antibiotics were incorporated into the TSB media at their respective minimal inhibitory concentrations: CHL at a concentration of 2 μg/mL, VAN at 1 μg/mL, and NOR at 1.25 μg/mL. Following the exposure to stress-inducing conditions, the *S. aureus* cultures were centrifuged at 15,928 × g for 3 min at 4°C. The cell pellets were resuspended in TSB and the resuspended cultures were inoculated at a concentration of 10^6^ CFU/mL in TSB treated with each antibiotic and incubated at 37°C and 120 rpm for 12 h. Subsequently, to confirm the antibiotic susceptibility change in *S. aureus* treated with the minimum inhibitory concentration (MIC) of antibiotics after exposure to stress and the resulting single-cell phenotypic alterations, 1-mL aliquots of the cultures were subjected to analysis using Raman spectroscopy and FCM.

**Table 1:** Antibiotics profiles and corresponding minimum inhibitor concentrations (MICs) for *Staphylococcus aureus* NCTC 8325–4.

**Antibiotics**	**Class**	**Mode of action**	**Target**	**MIC (mg/L)**	**Reference**
Vancomycin (VAN)	Glycopeptides and glycolipopeptides	Cell wall synthesis inhibitors	D-Ala-D-Ala moiety of NAM/NAG peptide subunits	1.0	Hiramatsu et al. (1997)
Chloramphenicol (CHL)	Chloramphenicol	Protein synthesis inhibitors	50S subunit of the ribosome and prevents the formation of peptide bonds	2	Stubbings et al. (2004)
Norfloxacin (NOR)	Quinolone	DNA gyrase	topoisomerase II and topoisomerase IV	1.25	Kaatz et al. (2005)

### 2.3 Raman spectroscopy

The bacterial cells were centrifuged at 15,928 × g for 5 min at 4°C and washed in phosphate-buffered saline (PBS). The samples were fixed with formaldehyde (4%, Sigma-Aldrich, St. Louis, MO, United States) for 2 h at 4°C in the dark and washed twice with cold PBS. A 2-μL drop of the sample was spotted on an aluminum-coated slide (LiMedlon Gmbh, Mannheim, Germany) and air-dried at 23°C. Single-cell Raman spectra (SCRS) were obtained using a Confocal Raman Imaging System (Nanobase, Seoul, Korea) equipped with an 1,800 g/mm grating, a 532 nm diode-pumped solid-state laser (Leading Tech, Shanghai, China), microscope body (Olympus, Tokyo, Japan), spectrometer (Nanobase, Seoul, Korea), and charge-coupled device (Atik Cameras, Bawburgh, United Kingdom). A 2-mW laser power was used for each single cell and the total acquisition time for each spectrum was 25 s. Twenty SCRS samples were collected from the formaldehyde-fixed cells. The SCRS were analyzed within the 400–1800 cm^−1^ range, and processed using the Chemospec package in R software (version 3.6.2; Hanson, 2015). The “baselineSpectra” function with “als” method was used to adjust the spectrum baseline using a second derivative constrained weighted regression. The “normSpectra” function was used with the method “Totlnt” to normalize the spectra by dividing the total intensity by the relative intensity.

### 2.4 Flow cytometry

A 1-mL volume of the cell suspension was acquired through centrifugation (15,928 × g, 5 min, 4°C). Reconstituted pellets were resuspended in 1 mL of sterile PBS at 4°C. The resuspended samples were diluted with PBS (1:100), stained using a LIVE/DEAD™ BacLight™ Bacterial Viability and Counting Kit (Invitrogen, Waltham, OR, United States), which contain SYTO 9 and propidium iodide (PI) for flow cytometry. Staining procedures followed manufacturer’s instructions. All samples were analyzed using a CytoFLEX flow cytometer (Beckman Coulter, Brea, CA, United States) equipped with two scatter detectors, five fluorescence detectors, and two lasers (blue: 488 nm and red: 638 nm). Fluorescence data were collected in the green [fluorescein isothiocyanate (FITC), 525/40 nm] and red [phycoerythrin (PE), 585/42 nm] channels, which are filters used to detect viable and dead cells, respectively. The events were collected for 1 min at a flow rate of 10 μL min^−1^. Total 30,000 cells were collected per sample to count viable cells and enumerate phenotypic diversity. Optical scattering, including forward scattering (FSC) and side scattering (SSC), facilitates the distinction between actual cells and noncellular particles. Subsequently, the cells were categorized into groups of viable, non-viable, and damaged cells, as determined by the density gradient within the fluorescein isothiocyanate-phycoerythrin (FITC-PE) biplot. During the denoising step, samples subjected to a 70% ethanol treatment for 30 min were introduced as a negative control. This served as a negative control, enabling precise cell gating on FITC-PE biplot and in distinguishing authentic cell data from non-cellular particles and dead cells. The distinct bacterial populations (live, dead and damaged cells) were gated based on the different viability stages in density plots using the R package “alphahull.” Total cell count (TCC) was measured by counting the cells in the cell-gating area. Depending on the different viability stages, the ratio was calculated by dividing the number of cells in each gated area by TTC. The phenotypic diversity was assessed by partitioning the denoised data from the FSC × FITC plot into 100 × 100 bins, based on previous research (Hong et al., 2021b). The R software (version 3.6.2) with identical scripts as those used in a previous study (Hong et al., 2021a) was used to process the data. The alpha diversity index (Shannon’s diversity index) and beta diversity were calculated using the R package “vegan” with the FCM feature tables.

### 2.5 Statistical analysis

All statistical analyses were performed using R software. The significance of the difference in phenotypic alpha diversity (D_a_) between the control and treated samples was calculated using a two-sample t-test, preceded by the Shapiro–Wilk test for normality assessment. The phenotypic beta diversity (D_b_) was depicted using a non-metric multidimensional scaling (NMDS) plot employing Bray–Curtis dissimilarity distances using the “vegan” R package. The analysis of similarity (ANOSIM) was carried out using the R package “vegan”-based function “anosim” to compute the significant difference between clusters represented in the NMDS plot.

Discriminant Analysis of Principal Components (DAPC) was employed to categorize SCRS according to the stressors encountered or antibiotics administered. This analysis was executed using the “dapc” function from the R package “adegenet,” which first transforms the data using principal component analysis and subsequently performs a discriminant analysis on the retained principal component via the “dudi.pca” function in the R package “ade4.” Subsequently, DA was performed on the retained principal components using the “lda” function in the “MASS” package. Statistical significance between the SCRS of the control and treated samples was assessed by conducting a two-sample t-test preceded by the Shapiro–Wilk test for normality assessment. Raman peaks demonstrating a value of *p* <0.01 were visualized in a heatmap.

## 3 Results

### 3.1 Effect of prior stressors on antibiotic resistance

**Figure 1:**
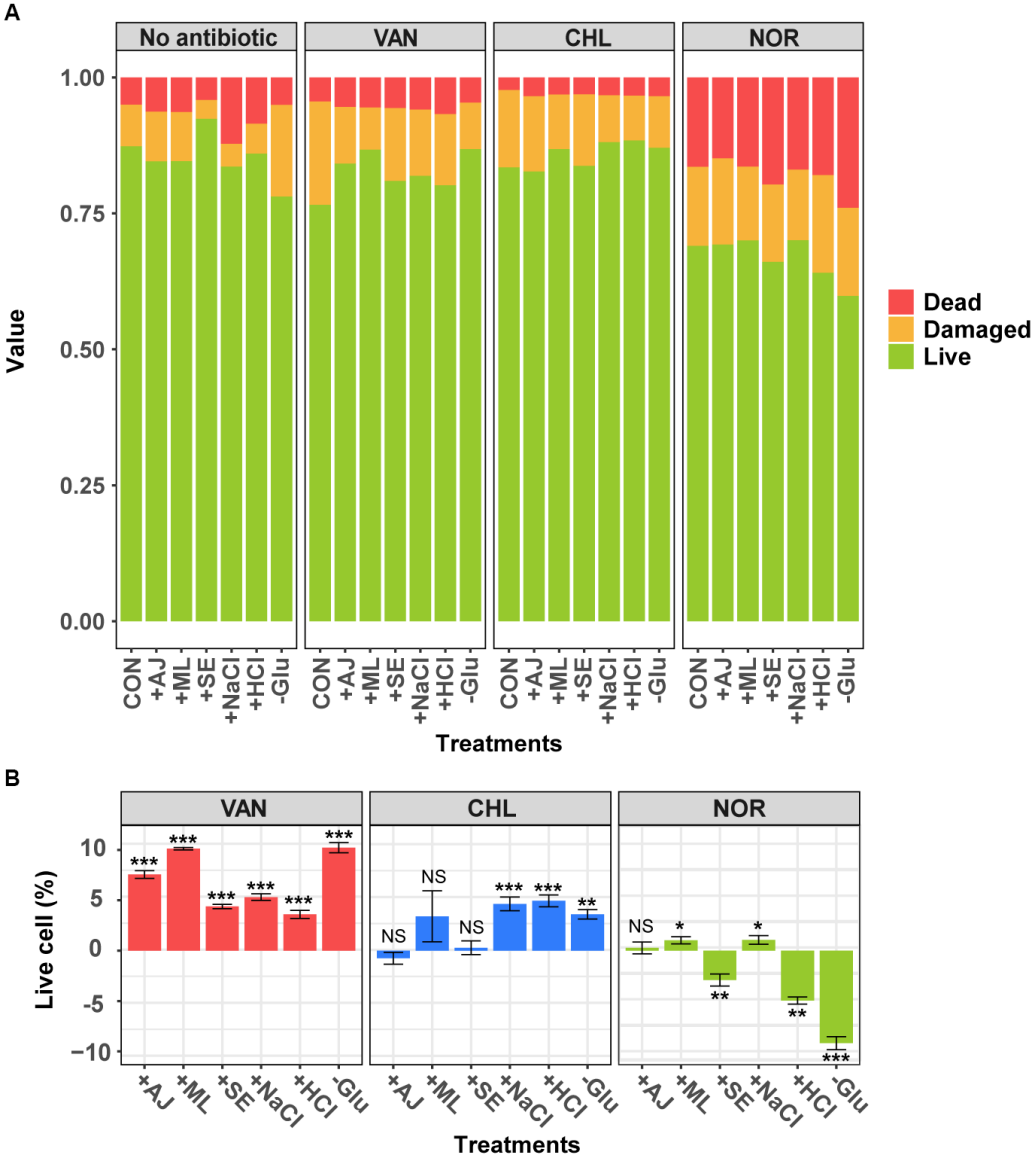
Analysis of cell viability in sample with antibiotics following pre-stress treatment using flow cytometry. **(A)** The change in the ratio of live, dead, and damaged cells according to the stress conditions and stress-exposed antibiotic treatments. **(B)** Change in the live cell percentage compared with each antibiotic-treated controls. Error bars represent the mean ± standard deviation (SD). The difference from the control was considered significant at *p*-value (NS, *p* > 0.05; *, *p* < 0.05; **, *p* < 0.01; ***, *p* < 0.001, *t*-test).

To examine the effects of prior stress on *S. aureus* growth, we assessed the cell viability of *S. aureus* in response to various stressors (Figure 1). In this study, all stressors except SE led to a significant reduction in the proportion of live cells compared to the control (Figure 1A; *t*-test, *p* < 0.05). The effects of the individual stressors on cell mortality and damage varied. AJ and ML elicited marginal increases in both dead and damaged cells, with percentages ranging from 0.1–1.1% and 0.5–1.0%, respectively. NaCl and HCl significantly increased the number of dead cells by 8.9 and 5.9%, respectively, while simultaneously reducing the number of damaged cells. Remarkably, exposure to Glu resulted in an 8.8% increase in the number of damaged cells, without affecting the proportion of dead cells. These results indicated that exposure to stressors for 12 h was sufficient to moderately reduce the proportion of live cells without causing acute growth inhibition in *S. aureus*. This level of stress appeared to be adequate for the conversion of *S. aureus* into a stress-adapted or stress-resistant phenotype.

To examine the effects of prior stress on antibiotic resistance in *S. aureus*, we assessed the viability of stressed *S. aureus* cells in response to antibiotics (Figure 1). The viability of the *S. aureus* cells varied significantly depending on the mode of action of the antibiotic. Cells treated with Glu and ML, in particular, showed a dramatic reduction in the number of damaged cells compared to the control (Figure 1A). Samples treated with VAN, which inhibits cell wall synthesis, showed an increase of 3.6–10.2% in live cells with enhanced antibiotic resistance (Figure 1B; *t*-test, *p* < 0.05). Interestingly, the biologically stressed samples showed no change in antibiotic resistance when treated with CHL. However, stressors containing NaCl, HCl, and Glu increased the number of live cells by approximately 4.4%. In contrast to the results of VAN and CHL treatments, samples treated with NOR, which inhibits DNA gyrase, showed that the stressors either maintained or reduced antibiotic resistance, resulting in a relatively higher proportion (−2.9 – −9.2%) of dead cells (Figures 1A,B). These results indicate that stress exposure in *S. aureus* promotes resistance to VAN, which primarily targets cell wall synthesis. However, stress-induced resistance is less effective against antibiotics with alternative mechanisms of action.

### 3.2 Effect of stressors or antibiotic treatment on phenotype diversity

**Figure 2:**
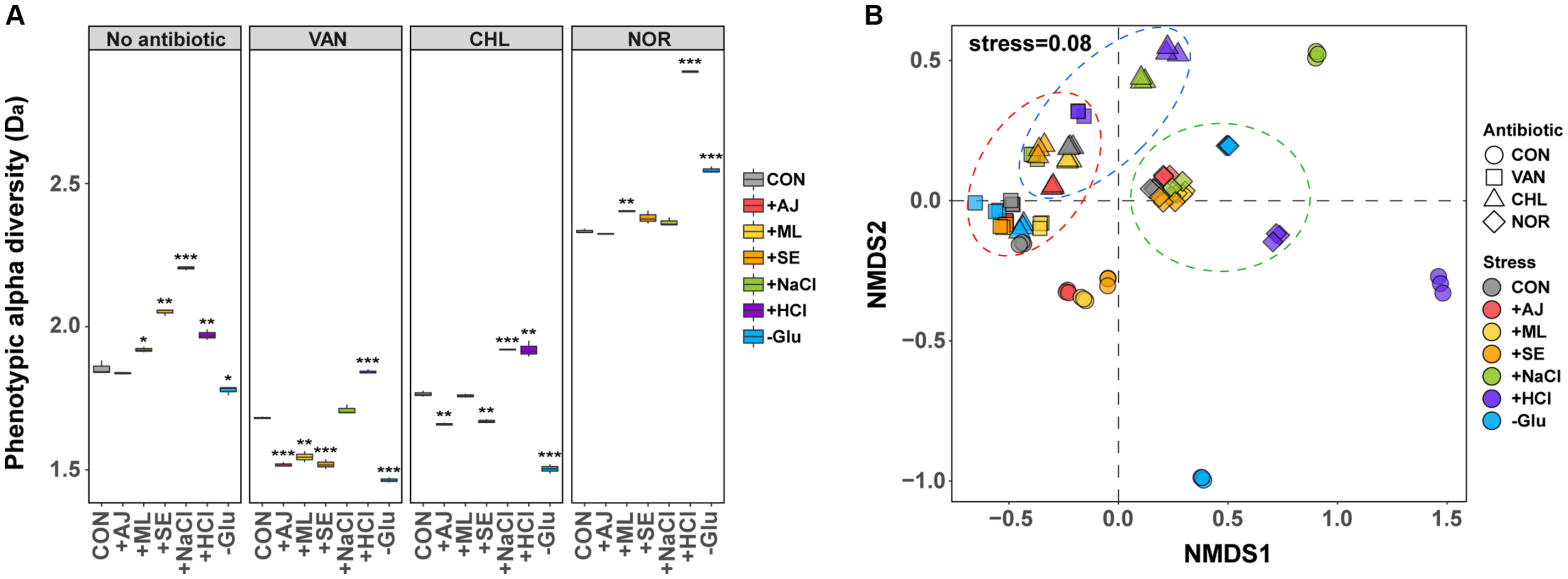
Differences in phenotype diversity between antibiotic treated control and stress-exposed antibiotic treatment by forward scattering (FSC)- fluorescein isothiocyanate (FITC) data from flow cytometry. **(A)** Box plots depict alpha diversities (D_a_) of live cell distribution by the Shannon index. *p*-values were annotated as follows: NS, *p* > 0.05; *, *p* < 0.05; **, *p* < 0.01; and ***, *p* < 0.001, *t*-test. **(B)** The phenotypic beta diversity (D_b_) analysis of the flow cytometry data separating samples according to the stress treatment and stress-exposed antibiotic treatment by non-metric multidimensional scaling (NMDS) based on the Bray–Curtis distance metric.

To quantitatively compare changes in phenotypes of *S. aureus* because of stress exposure or antibiotic treatment, we compared phenotypic D_a_ calculated using FCM. The D_a_ of *S. aureus* exposed to all the stressors was significantly altered, except for AJ (Figure 2A). The NaCl-treated *S. aureus* (2.2 ± 0.01) showed the largest increase in D_a_ compared to the control (1.9 ± 0.03), followed by HCL, SE, and ML. GLU was an exception, with reduced D_a_ (t-test, *p* < 0.05). These results suggest that *S. aureus* changes its phenotype within 12 h of exposure to stressors.

Antibiotic treatment led to a notable modification in the D_a_ of *S. aureus*, depending on the specific mechanism of action of the antibiotics used (Figure 2A). For controls treated with VAN (1.7 ± 0.01) and CHL (1.8 ± 0.01), a marginal reduction in D_a_ was observed compared to the untreated samples (1.9 ± 0.03). Conversely, the controls subjected to NOR treatment (2.3 ± 0.01) exhibited a substantial increase in D_a_. When stressed by microbial interactions (AJ, ML, and SE) and subjected to VAN and CHL treatment, D_a_ was significantly reduced by 4.9–18.0% compared to the control (*t*-test, *p* < 0.05). Glu showed a much more dramatic decrease in Da (18.7–20.9%), whereas NaCl and HCl both increased compared to the control. All cultures of *S. aureus* treated with NOR showed an increase in D_a_ compared to the control when stressed, with a particularly noticeable increase in the HCl-(2.9 ± 0.01) and Glu-treated (2.6 ± 0.01) samples (*t*-test, *p* < 0.05). These findings suggest that, even when subjected to equivalent stress conditions, the tendency for phenotypic alterations in *S. aureus* differs substantially based on the mode of action of the specific antibiotic.

To assess variations in phenotypic characteristics influenced by stressors or antibiotic treatments, an nMDS approach was employed to evaluate phenotypic beta diversity (D_b_, Figure 2B). Phenotypic alterations in *S. aureus* induced by the stressors exhibited marked deviations from those observed in the control group (ANOSIM, *p* < 0.05). Notably, changes induced by microbial interactions were tightly clustered, yet distinct from the control, whereas phenotypes influenced by the other three types of stressors were clearly disparate from the control and unique to each stressor. These findings imply that the phenotypic responses of *S. aureus* result in stress-specific phenotypic outcomes.

Although *S. aureus* manifested stress-specific phenotypes, these phenotypes tended to converge notably when subjected to subsequent antibiotic treatment (ANOSIM, p < 0.05). Specifically, the phenotype under the NOR treatment mostly clustered tightly with that of the unstressed control, with notable exceptions for HCl and Glu. *S. aureus* pre-exposed to microbial interactions exhibited a phenotype resembling that of the control when treated with VAN or CHL. However, *S. aureus* subjected to NaCl, HCl, and Glu treatments showed considerable divergence from the control phenotype. These observations suggest that antibiotics exert a considerably greater influence on phenotypic modifications than stressors and that the extent of phenotypic alteration prior to antibiotic treatment is generally sustained during antibiotic treatment.

### 3.3 Effect of stressors on the biomolecular phenotypes

**Figure 3:**
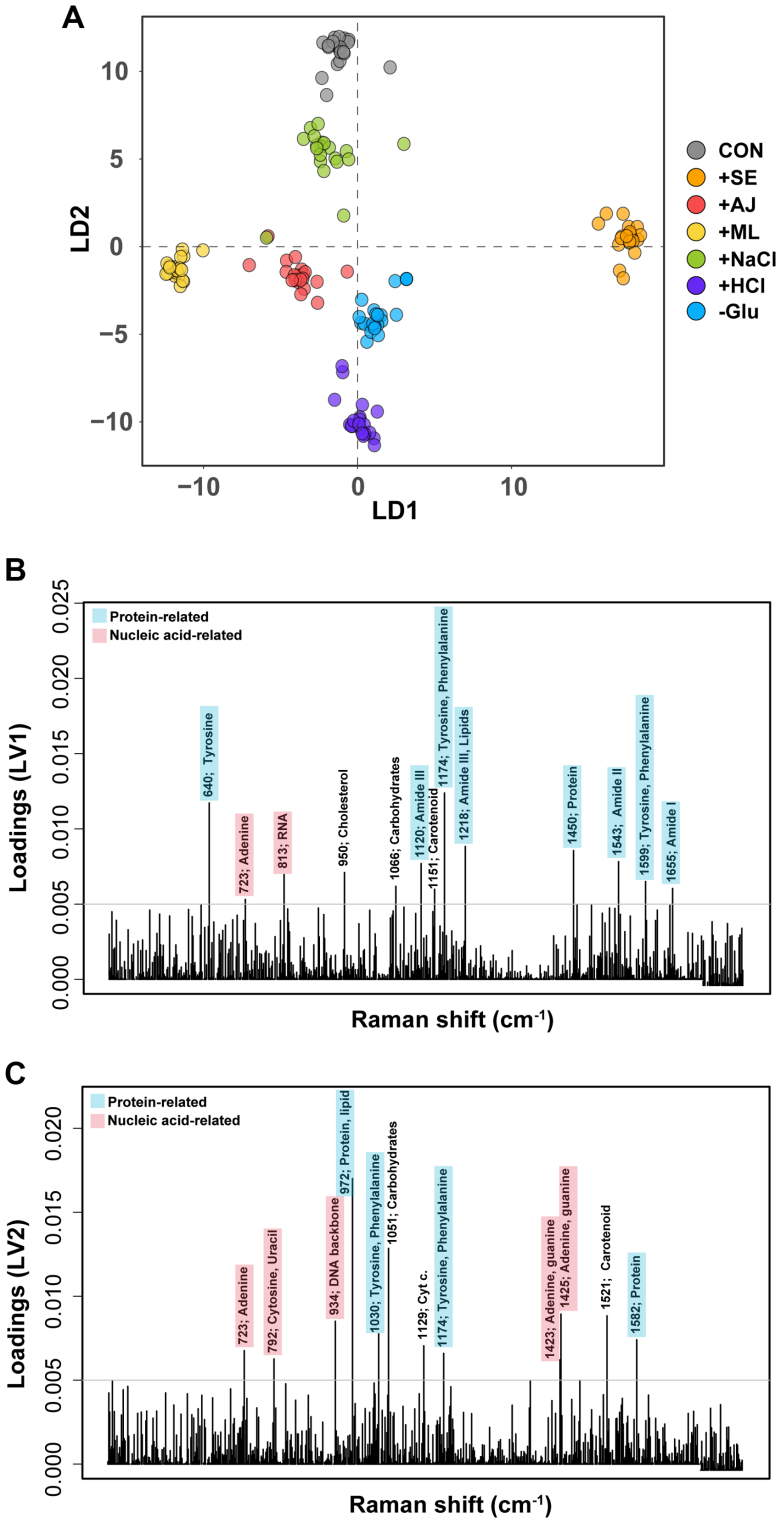
Discriminant of *Staphylococcus aureus* cultures according to the stress treatments. **(A)** Discriminant Analysis of Principal Components (DAPC) plot of exclusively stress treatments (antibiotic non-treatment). **(B)** Contributions of Raman peak to the first principal component of the DAPC of exclusively stress-treated sample data. **(C)** Contributions of Raman peak to the second principal component of the DAPC of exclusively stress-treated sample data. Only Raman peak whose contribution is above an arbitrary threshold (grey horizontal line) are indicated for clarity.

To evaluate the biomolecular phenotypic shifts in *S. aureus* induced by stressors or antibiotics, SCRS was obtained for individual samples and subsequently compared (Supplementary Figure S1). DAPC modeling revealed discernible spectral distinctions among various stressors (Figure 3A). Interestingly, phenotypic alterations induced by microbial interactions were distinctly represented in the DAPC plot, with Linear Discriminant 1 (LD1) serving as the reference for the control group and LD2 delineating the effects of other stressors. Furthermore, we extracted the most contributing features associated with each LD, revealing the primary phenotypic alterations induced by stressors at the biomolecular level. They were dominated by the vibrational features of proteins and nucleic acids from SCRS (Figures 3B,C). The LD1 loading contained Raman peaks for proteins (640 cm^−1^ for tyrosine, 1,120 cm^−1^ for amide III, 1174 cm^−1^ for tyrosine and phenylalanine, 1,218 cm^−1^ for amide III and lipid, 1,450 cm^−1^ for protein, 1,543 cm^−1^ for amide II, 1599 cm^−1^ for tyrosine and phenylalanine, and 1,655 cm^−1^ for amide I) and nucleic acids (723 cm^−1^ for adenine and 813 cm^−1^ for RNA) (Figure 3B). The loading on LD2 captured Raman peaks different from those in the LD1 layers, except at 723 and 1,174 cm^−1^. Loading on LV2 contained proteins (972 cm^−1^ for proteins and lipids, 1,030 cm^−1^ for tyrosine and phenylalanine, and 1,582 cm^−1^ from proteins) and nucleic acids (792 cm^−1^ from cytosine and uracil, 934 cm^−1^ from the DNA backbone, 1,423 cm^−1^ from adenine and guanine, and 1,425 cm^−1^ from adenine and guanine).

### 3.4 Impact of stressors on antibiotic-treated phenotypes

**Figure 4:**
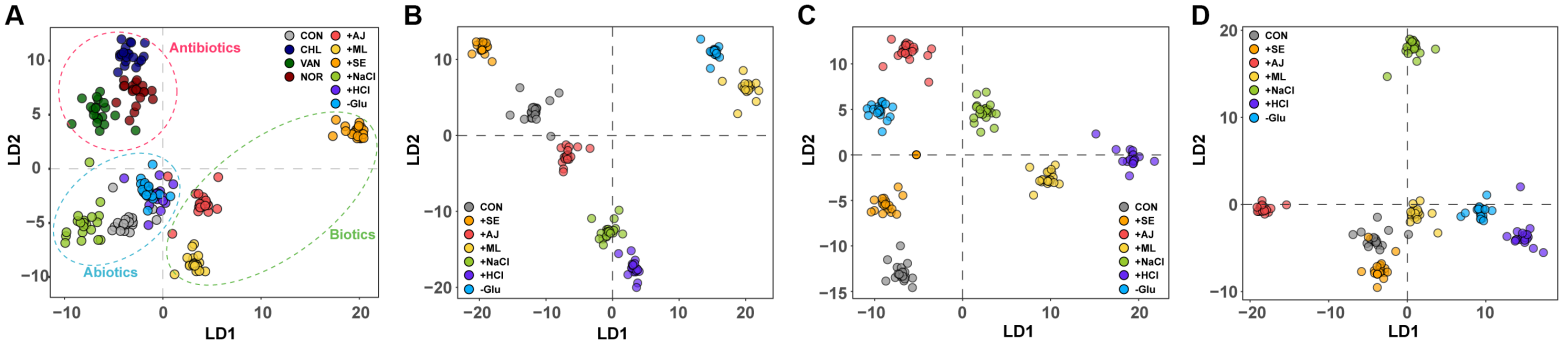
Discriminant of *S. aureus* cultures according to the antibiotic treatment of stress-exposed samples. **(A)** DAPC plot of Single-cell Raman spectra (SCRS) showing the difference between exclusively antibiotic-treated samples and exclusively stress-exposed samples **(B)** stress-exposed VAN treatment, **(C)** stress-exposed CHL treatment, and **(D)** stress-exposed NOR treatment.

Raman spectroscopy was also used to examine the biomolecular phenotypic shift in *S. aureus* cells that had been pre-stressed following antibiotic treatment. In the DAPC plot, cells treated with the three distinct antibiotics, each with a unique mode of action, formed more closed clusters (Figure 4A). Interestingly, the cells subjected to antibiotic treatment exhibited phenotypic profiles that were more similar to each other than those exposed to individual stressors (Figure 4A). This observation implies that the impact of antibiotics on the phenotypic characteristics of *S. aureus* cells is notably more pronounced than that of the stressors.

To assess the impact of pre-stress conditions on the phenotypes of *S. aureus* cells subjected to antibiotic treatment, we compared sample-specific SCRS with individual antibiotics. Irrespective of the class of antibiotics used, pre-stressed cells manifested distinct phenotypic profiles in comparison with control cells that were solely exposed to antibiotics (Figures 4B–D). Furthermore, a differential phenotypic deviation from the control group was observed in cells initially exposed to abiotic stressors such as NaCl, HCl, and Glu, compared to cells involved in microbial interactions. This suggests that the phenotype altered by the stressor influences phenotypic differentiation following subsequent antibiotic treatment.

**Figure 5:**
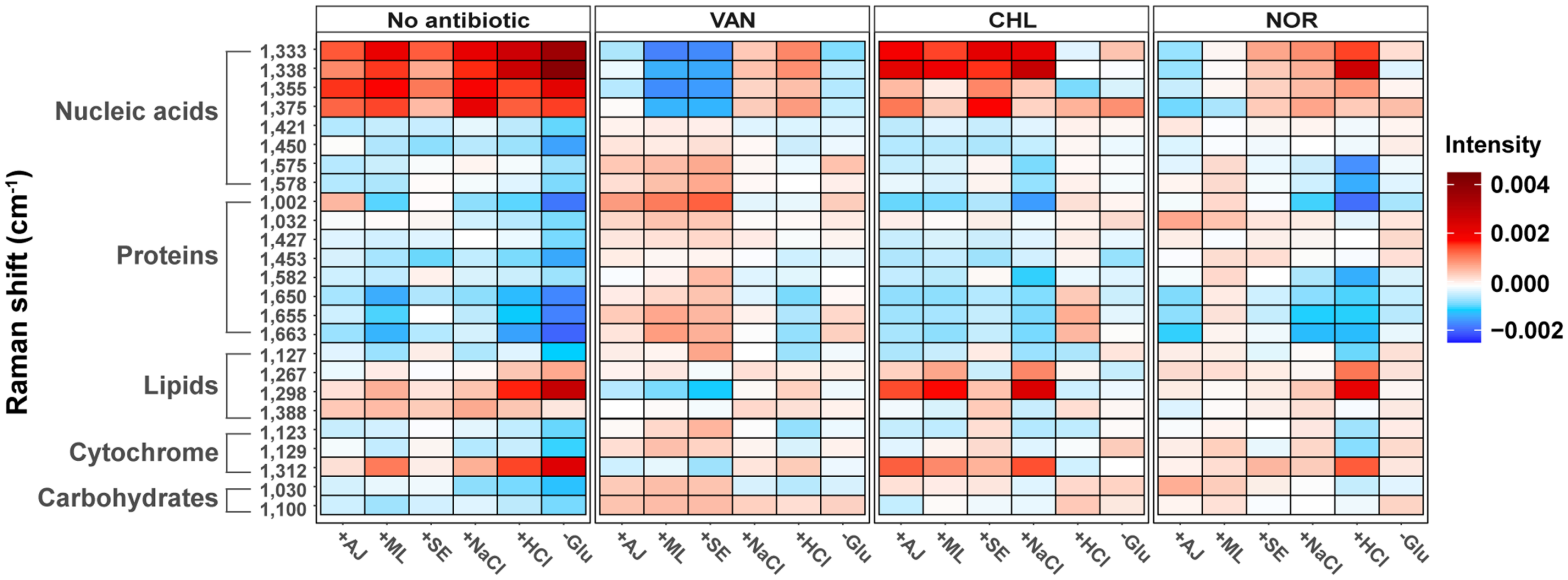
Changes in bacterial biochemical composition with various stress treatments and stress-exposed different antibiotic treatments using the Raman spectrum. Heat maps were generated to show the peak intensity trends according to the stress treatments and stress-exposed antibiotic treatments. The mean spectral intensity for each stress-exposed antibiotic treatments sample was colored by subtracting the average spectral intensity for each antibiotic treatment of the control group.

To elucidate the effects of stressors or antibiotics on the biomolecular phenotype, we generated heat maps illustrating variations in Raman peaks corresponding to nucleic acids, proteins, lipids, carbohydrates, and cytochromes (Figure 5 and Supplementary Table S1). Irrespective of the specific stressor applied, consistent elevation was observed in the Raman peaks corresponding to a variety of nucleic acids (1,333 cm^−1^, 1,338 cm^−1^, 1,355 cm^−1^, and 1,375 cm^−1^), as well as those linked to cytochromes (1,312 cm^−1^) and lipids (1,267 cm^−1^, 1,298 cm^−1^, and 1,388 cm^−1^). The magnitude of the increase in Raman intensity for the elevated biomolecules varied in response to stressors. Furthermore, certain Raman peaks, including 1,002 cm^−1^ (protein), 1,032 cm^−1^ (protein), and 1,127 cm^−1^ (lipid), showed both elevations and reductions in Raman intensity according to the type of stressor applied.

In the case of VAN, *S. aureus* cells with prior microbial interactions displayed elevated Raman intensities in peaks corresponding to proteins and carbohydrates relative to control samples (Figure 5). In contrast, these cells exhibited a marked reduction in Raman peaks associated with partial nucleic acids. Conversely, cells that were pre-exposed to NaCl and HCl manifested a contrasting trend; specifically, a pronounced increase in the Raman peaks corresponding to the same nucleic acids that decreased under microbial stress. These results underscore the notion that even when subjected to identical antibiotic treatments, the biomolecular profile of *S. aureus* cells can exhibit substantial variations in Raman intensity depending on the preceding stressors. In most CHL-treated samples, a substantial increase was observed in the Raman peaks corresponding to nucleic acids and lipids, whereas a decline was noted in the protein-related peaks (Figure 5). Conversely, the Raman profiles of the samples pretreated with HCl and Glu remained largely consistent with those of the control group. In contrast to the pronounced effects observed under the VAN and CHL treatments, most stressors had a negligible impact on the biomolecular phenotype of *S. aureus* cells when treated with NOR.

### 3.5 Phenotypic changes associated with antibiotic resistances

**Figure 6:**
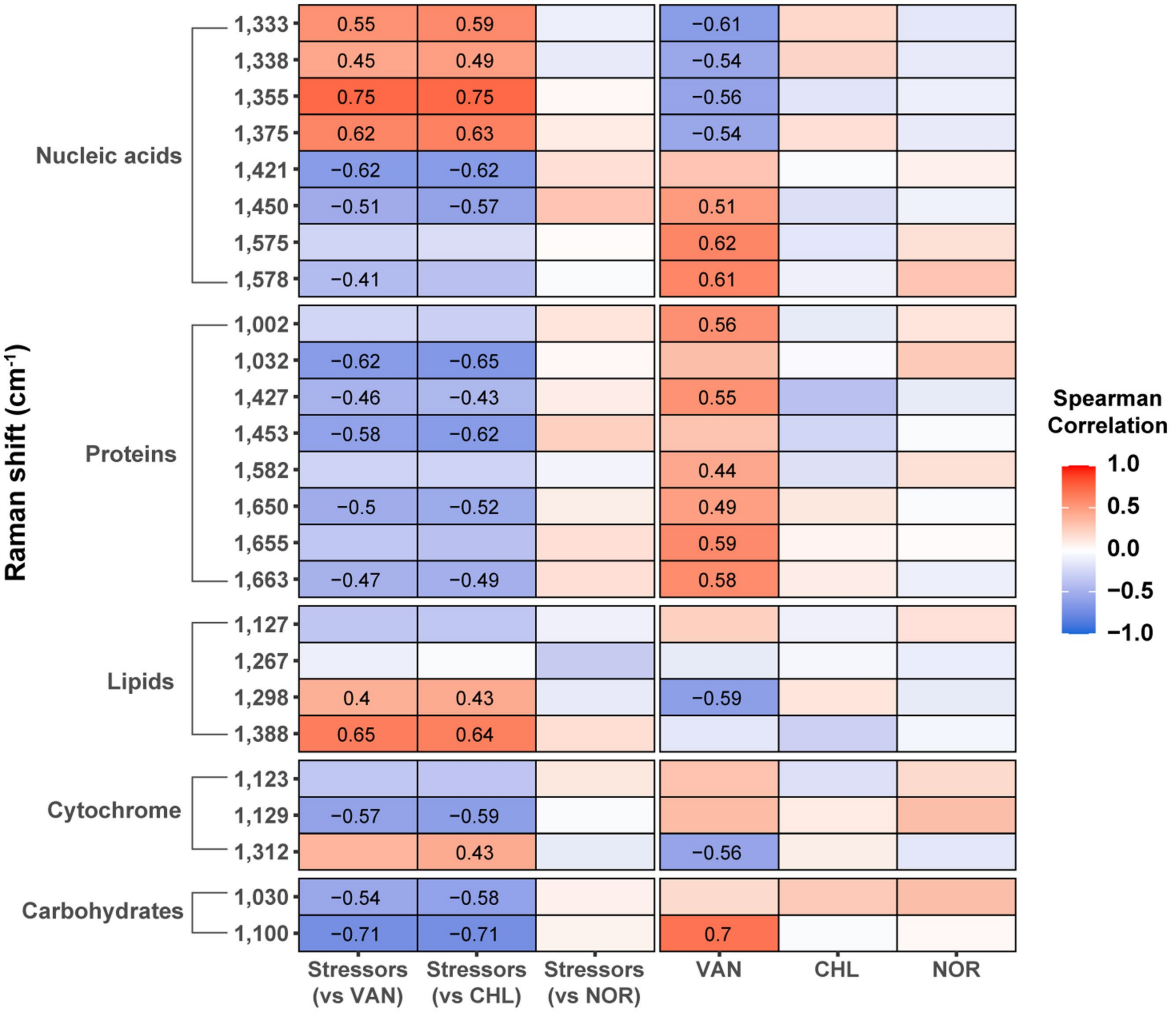
Correlation between the increase in live cell percentage after antibiotic treatment and Raman peak intensity. The numbers inside the box represent the Spearman correlation coefficient with *p*-value <0.05.

To determine the association between stressor- or antibiotic-altered phenotypes and antibiotic resistance, we performed a correlation analysis between the proportion of live cells with antibiotic resistance and Raman intensity after stress or antibiotic treatment (Figure 6). Raman peaks related to lipids (1,298 cm^−1^ and 1,388 cm^−1^), and nucleic acids (1,333 cm^−1^, 1,338 cm^−1^, 1,355 cm^−1^, and 1,375 cm^−1^) in *S. aureus* that were increased by stressors were positively correlated with resistance to VAN and CHL (*p* < 0.05). A substantial number of other Raman peaks negatively correlated with antibiotic resistance (*p* < 0.05). Stress-induced phenotypes were not associated with resistance to NOR at all. These results indicate that stressor-induced changes in specific biomolecular phenotypes are strongly associated with increased antibiotic resistance of specific antibiotics. An opposite relationship was identified between the phenotype of *S. aureus* treated with VAN and increased resistance to antibiotics, further confirming the influence of prior stressors on VAN resistance. For CHL and NOR, which did not show a clear trend toward stress-induced resistance, it was difficult to find a significant association between stressor- and antibiotic-induced phenotypic changes and resistance.

## 4 Discussion

The growing issue of antibiotic resistance of *S. aureus* warrants critical attention, given its symbiotic relationship with humans (Poudel et al., 2023). Previous research has primarily focused on the antibiotic resistance of *S. aureus* under stress conditions, namely salinity, pH, and microbial interaction (Lemaire et al., 2007; Vaish et al., 2018; Nabb et al., 2019). In nature, a microbe frequently encounters stressors prior to antibiotic treatment, a factor that prior research approaches have incompletely accounted for in characterizing the behavior of antibiotic resistance in *S. aureus*. This study offers novel contributions by analyzing the response of *S. aureus* cells to antibiotics under pre-existing stress conditions, during which the cells exhibit altered phenotypes. Previous studies have presented a controversial narrative on the role of environmental stressors in shaping antibiotic resistance, with some indicating the upregulation and others indicating the downregulation of resistance attributes (Foster, 2017). Our results align with the ongoing debate and provide empirical evidence emphasizing the role of antibiotic class in modulating resistance, particularly noting that resistance to VAN is promoted under pre-stress conditions. Conversely, under the influence of NOR, we observed a predominantly declining pattern of antibiotic resistance with a mixed response in the context of CHL. Therefore, the mechanistic pathway underlying antibiotic resistance in *S. aureus* appears to be closely associated with the specific environmental stressors and antibiotics used, enriching our understanding of the versatility of antibiotic resistance mechanisms.

All bacterial species employ ubiquitous defense strategies in response to environmental stressors, resulting in phenotypic modifications (Tan et al., 2022; Wee et al., 2022). These altered phenotypes are transiently maintained as part of adaptive or evolutionary processes (Horton et al., 2023). Therefore, we hypothesized that phenotypic alterations induced by stressors are associated with elevated antibiotic resistance. In this context, we first quantified these changes using phenotypic diversity (D_a_ and D_b_), which revealed that different stressors led to distinct phenotypic diversification. This highlights the ability of *S. aureus* to undergo rapid phenotypic shifts in response to environmental stresses. Interestingly, exposure to antibiotics further diversified these phenotypic alterations, depending on the mode of action of the antibiotics. These results suggest that bacterial cells respond differently to antibiotics depending on their phenotype or readiness to respond. At the single cell level within an identical population, a high degree of phenotypic and transcriptional heterogeneity exists (Lee et al., 2014). This complexity is observed in natural environments as well as in controlled *in vitro* laboratory experiments. These individual phenotypic or transcriptional differences can lead to subtle differences in the efficient response of individual cells to antibiotics, some of which can promote antibiotic resistance beyond the usual defense mechanisms against antibiotics (Davies and Davies, 2010; Peterson and Kaur, 2018). While individual differences in antibiotic resistance can be attributed to individual genetics or differences in the pathogen that a person carries, it is also possible that the pathogenic phenotype induced by the environment to which a person is exposed can lead to the promotion of antibiotic resistance. This is of critical importance as it suggests that therapeutic outcomes could be modulated by prior environmental stressors, a variable often overlooked in antibiotic treatments.

Employing SCRS and DAPC modeling, this study provides novel insights into the biomolecular underpinnings of phenotypic shifts caused by stressors or antibiotics. Stressors and antibiotics can induce significant changes in the vibrational features of proteins, lipids, and nucleic acids. This biomolecular remodeling underscores the complex regulatory networks initiated in response to environmental stressors, thereby influencing antibiotic resistance (Kik et al., 2023). These regulatory networks often involve complex interactions between transcription factors, signaling molecules, and effector proteins, resulting in a cascade of events that can ultimately lead to phenotypic changes. Such changes may include modifications in membrane permeability, the activation of efflux pumps, and even the secretion of enzymes that can degrade antibiotics, thus enhancing microbial survival in hostile environments (Sánchez-Romero and Casadesús, 2014; Frenoy and Bonhoeffer, 2018). Our findings confirmed that pre-stress conditions markedly influenced the phenotypic outcomes of nucleic acids and lipids upon subsequent antibiotic treatment. These results signify that the initial stress environment can “prime” bacterial cells in a manner that is consequential to subsequent antibiotic treatments. This has wide-ranging implications, suggesting that the “history” of environmental exposure could be an important parameter in determining the effectiveness of antibiotic treatments, potentially necessitating the re-evaluation of therapeutic strategies.

We identified significant associations between specific biomolecular markers and antibiotic resistance, as evidenced by elevated Raman peaks corresponding to lipids, and nucleic acids, and an increase in live cell proportions after VAN and CHL treatment. These results are consistent with transcriptomics in previous study. Significant correlations between spectral peak intensities and the expression of antibiotic resistance genes indicate that metabolic shifts associated with resistance are detectable both in Raman spectral signatures and gene expression profiles (Germond et al., 2018). The implications of this observation are manifold and extend to the development and assessment of antibiotics. By understanding the relationship between stress-induced biomolecular markers and antibiotic resistance, we propose the feasibility of developing predictive models of antibiotic resistance patterns. Such predictive capabilities can be invaluable for monitoring the effectiveness of antibiotic therapies in real time and informing the design of next-generation antibiotics that can effectively counteract stressor-induced resistance mechanisms. Ultimately, this insight will contribute to the development of more robust and effective strategies for mitigating antibiotic-resistant infections, with significant implications for public health and clinical management.

This study offers valuable insights into the effects of different stressors on the phenotype and antibiotic resistance characteristics of *S. aureus*. However, this study had several limitations that warrant further discussion. First, the observed changes in antibiotic resistance and phenotypic diversity were assessed within a 12-h exposure, limiting the applicability of the results to chronic or long-term stress conditions. Previous reports have highlighted the dynamic nature of microbial stress responses and resistance mechanisms over extended periods, presenting a temporal dimension not captured in this study (Tan et al., 2022). Secondly, the experimental design did not incorporate multiple bacterial strains or antibiotics, thus restricting the generalizability of the findings. Employing a wider array of antibiotics and strains can provide a more comprehensive view of bacterial adaptability. For instance, using a SigB proficient strain that is highly resistant to environmental stress, as opposed to the SigB-impaired strain used in this study, would allow for a broader spectrum of stress-induced antibiotic resistance. Finally, although this work employed advanced techniques, such as Raman spectroscopy and FCM, cross-validation with other methodological approaches, including transcriptomics or proteomics, was absent. To address these gaps, future studies should consider a longitudinal design encompassing multiple bacterial strains, integrate cs ‘omics’ technologies for mechanistic insights, and employ complementary analytical techniques for robust validation.

In conclusion, this study reveals a complex relationship between environmental stressors and antibiotic resistance in *S. aureus*. By employing Raman spectroscopy and FCM, this study not only validated the phenotypic diversification arising from stress conditions but also highlighted the antibiotic class-specific modulation of resistance. Furthermore, this study underscores the role of pre-stress conditions in “priming” bacterial cells, thereby affecting the efficacy of subsequent antibiotic treatments. These findings have critical implications for treatment regimens for antibiotic-resistant infections, suggesting that prior environmental contexts could affect therapeutic outcomes. Furthermore, the study enabled the identification of key biomolecular markers associated with antibiotic resistance, offering avenues for the development of predictive models that could inform next-generation antibiotic therapeutic regimens. Overall, these results contribute significantly to our understanding of antibiotic resistance mechanisms and provide insights that could be transformative for both clinical practice and public health strategies.

## Data availability statement

The data presented in this study were deposited in FlowRepository (accession number FR-FCM-Z6RS).

## Author contributions

GW: Conceptualization, Data curation, Investigation, Methodology, Validation, Visualization, Writing – original draft, Writing – review & editing. EL: Data curation, Methodology, Writing – review & editing. SN: Data curation, Validation, Writing – review & editing. TL: Conceptualization, Supervision, Writing – original draft, Writing – review & editing.
