# Supplementary material for: Phenotypic shifts induced by environmental pre-stressors modify antibiotic resistance in Staphylococcus aureus
Source: Front Microbiol. 2023 Dec 4;14:1304509. doi: 10.3389/fmicb.2023.1304509 (PMC10725907; doi:10.3389/fmicb.2023.1304509)
Supplement: Supplementary file 1 [file Data_Sheet_1.docx]

Supplementary Material

Phenotypic shifts induced by prior stressors alter the antibiotic resistance of *Staphylococcus aureus*

**Gui Nam Wee a,b, Eun Sun Lyou a, Susmita Das Nishu a, Tae Kwon Leea,**

*** Correspondence:** Tae Kwon Lee : tklee@yonsei.ac.kr


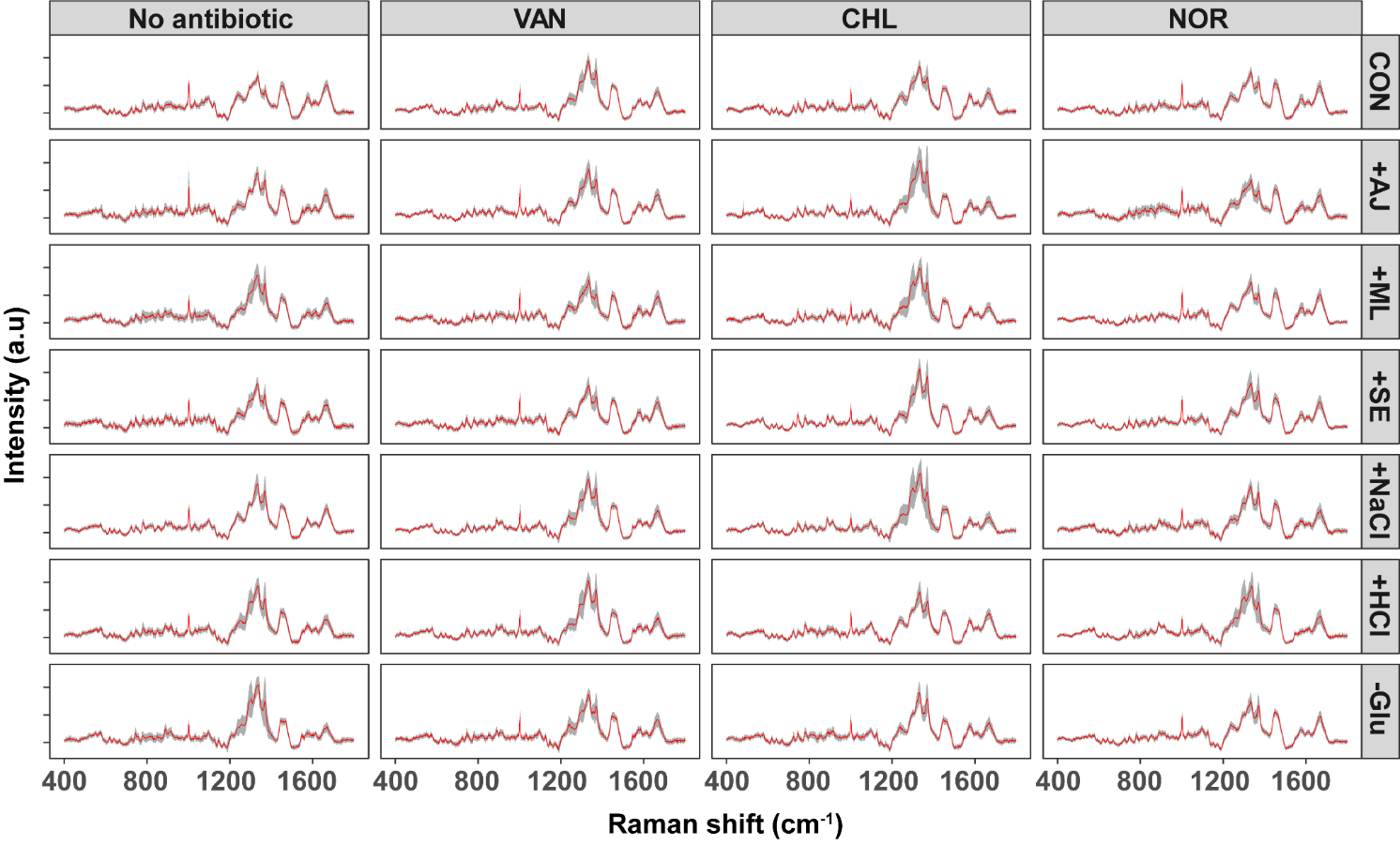
**Supplementary Figure 1.** Single-cell Raman spectra (SCRS) of *Staphylococcus aureus* according to the stress treatments and stress-exposed antibiotic treatments. SCRS of 20 cells were averaged. The solid lines represent average of SCRS from individual cells and the grey shadow represents standard deviation of SCRS. CON; control.

**Supplementary Table 1.** Raman frequency of stress-exposed antibiotic treatment samples with significantly different intensities compared to stress non-treated samples.

| **Raman shift (cm^-1^)** | **Assignment** | **Group** | **Reference** |
| --- | --- | --- | --- |
| 1,002 | Phenylalanine | Phenylalanine, b-carotene | (Strola et al., 2014) |
| 1,030 | -C-C-skeletal), C-O, def(C-O-H) | Carbohydrates | (Schuster et al., 2000) |
| 1,032 | C-N str | Phenylalanine | (Huang et al., 2010) |
| 1,100 | -C-C-skeletal), C-O, def(C-O-H) | Carbohydrates | (Schuster et al., 2000) |
| 1,123 | CH Phe | Cytochrome | (Notingher and Hench, 2006) |
| 1,127 | =C-C= (unsaturated fatty acids in lipids) | lipids | (Huang, et al., 2010) |
| 1,129 | ν(C−N) | Cyt c. | (Maquelin et al., 2002) |
| 1,267 | Lipids | Lipids | (van Manen et al., 2005) |
| 1,298 | CH_2_twist. | Saturated lipid | (Notingher and Hench, 2006) |
| 1,312 | δ(C−H) | Cyt c. | (Cui et al., 2018) |
| 1,333 | CH_3_CH_2_ def. of collagen | Nucleic acid, protein | (Teng et al., 2016) |
| 1,338 | Adenine, guanine, tryrosine, tryptophan | Adenine, guanine, tryrosine, tryptophan | (Strola et al., 2014) |
| 1,355 | A, G, CH def. | Nucleic acid, protein | (Notingher and Hench, 2006) |
| 1,375 | Thymine, adenine, guanine | Thymine, adenine, guanine | (Uzunbajakava et al., 2003) |
| 1,388 | CH_3_ | Lipid | (Teng et al., 2016) |
| 1,421 | Adenine, guanine | Adenine, guanine | (Uzunbajakava et al., 2003) |
| 1,427 | Adenine, guanine | Adenine, guanine | (Uzunbajakava et al., 2003) |
| 1,450 | G, A, CH def. | Nucleic acid, protein, lipid, carbohydrate | (Notingher and Hench, 2006) |
| 1,453 | Protein | Protein | (Huang et al., 2010) |
| 1,575 | Guanine, adenine (ring str) | Guanine, adenine | (Maquelin et al., 2002) |
| 1,578 | Adenine, cytosine, guanine | Nucleic acid | (Uzunbajakava et al., 2003) |
| 1,582 | Protein | Protein | (Maquelin et al., 2002) |
| 1,650 | Amide I | Amide I | (Maquelin et al., 2002) |
| 1,655 | Amide I | Amide I | (Strola et al., 2014) |
| 1,663 | Amide I | Amide I | (Maquelin et al., 2002) |

**Reference**

Cui, L., Yang, K., Li, H.-Z., Zhang, H., Su, J.-Q., Paraskevaidi, M., et al. (2018). Functional Single-Cell Approach to Probing Nitrogen-Fixing Bacteria in Soil Communities by Resonance Raman Spectroscopy with 15N2 Labeling. *Analytical Chemistry* 90(8)**,** 5082-5089. doi: 10.1021/acs.analchem.7b05080.

Huang, W.E., Li, M., Jarvis, R.M., Goodacre, R., and Banwart, S.A. (2010). Chapter 5 - Shining Light on the Microbial World: The Application of Raman Microspectroscopy. *Advances in Applied Microbiology* 70**,** 153-186. doi: 10.1016/S0065-2164(10)70005-8.

Maquelin, K., Kirschner, C., Choo-Smith, L.P., van den Braak, N., Endtz, H.P., Naumann, D., et al. (2002). Identification of medically relevant microorganisms by vibrational spectroscopy. *Journal of Microbiological Methods* 51(3)**,** 255-271. doi: 10.1016/s0167-7012(02)00127-6.

Notingher, I., and Hench, L.L. (2006). Raman microspectroscopy: a noninvasive tool for studies of individual living cells in vitro. *Expert Review of Medical Devices* 3(2)**,** 215-234. doi: 10.1586/17434440.3.2.215.

Schuster, K.C., Urlaub, E., and Gapes, J.R. (2000). Single-cell analysis of bacteria by Raman microscopy: spectral information on the chemical composition of cells and on the heterogeneity in a culture. *Journal of Microbiological Methods* 42(1)**,** 29-38. doi: 10.1016/S0167-7012(00)00169-X.

Strola, S.A., Marcoux, P.R., Schultz, E., Perenon, R., Simon, A.-C., Espagnon, I., et al. (2014). Differentiating the growth phases of single bacteria using Raman spectroscopy. *SPIE BiOS* 8939. doi: 10.1117/12.2041446.

Teng, L., Wang, X., Wang, X., Gou, H., Ren, L., Wang, T., et al. (2016). Label-free, rapid and quantitative phenotyping of stress response in E. coli via ramanome. *Scientific Reports* 6(1)**,** 34359. doi: 10.1038/srep34359.

Uzunbajakava, N., Lenferink, A., Kraan, Y., Volokhina, E., Vrensen, G., Greve, J., et al. (2003). Nonresonant confocal Raman imaging of DNA and protein distribution in apoptotic cells. *Biophys J* 84(6)**,** 3968-3981. doi: 10.1016/s0006-3495(03)75124-8.

van Manen, H.J., Kraan, Y.M., Roos, D., and Otto, C. (2005). Single-cell Raman and fluorescence microscopy reveal the association of lipid bodies with phagosomes in leukocytes. *Proc Natl Acad Sci U S A* 102(29)**,** 10159-10164. doi: 10.1073/pnas.0502746102.
